# Muscle force distribution of the lower limbs during walking in diabetic individuals with and without polyneuropathy

**DOI:** 10.1186/s12984-017-0327-x

**Published:** 2017-11-09

**Authors:** Aline A. Gomes, Marko Ackermann, Jean P. Ferreira, Maria Isabel V. Orselli, Isabel C. N. Sacco

**Affiliations:** 1 Physical Education and Physiotherapy Faculty, Federal University of Amazonas, Manaus, AM Brazil; 2 Department of Mechanical Engineering, FEI University, Sao Bernardo do Campo, SP Brazil; 3 Department of Physical Therapy, Federal University of Sao Carlos, Sao Carlos, SP Brazil; 4 Biomedical Engineering Course, Franciscan University, Santa Maria, RS Brazil; 5 Physical Therapy, Speech and Occupational Therapy department, School of Medicine, University of Sao Paulo, Sao Paulo, SP Brazil; 6 Centro de Docência e Pesquisa do Departamento de Fisioterapia, Fonoaudiologia e Terapia Ocupacional, Rua Cipotânea, 51, Cidade Universitária, São Paulo, SP CEP: 05360-160 Brasil

**Keywords:** Biomechanics, Gait, Computer simulation, Diabetic neuropathies

## Abstract

**Background:**

Muscle force estimation could advance the comprehension of the neuromuscular strategies that diabetic patients adopt to preserve walking ability, which guarantees their independence as they deal with their neural and muscular impairments due to diabetes and neuropathy. In this study, the lower limb’s muscle force distribution during gait was estimated and compared in diabetic patients with and without polyneuropathy.

**Methods:**

Thirty individuals were evaluated in a cross-sectional study, equally divided among controls (CG) and diabetic patients with (DNG) and without (DG) polyneuropathy. The acquired ground reaction forces and kinematic data were used as input variables for a scaled musculoskeletal model in the OpenSim software. The maximum isometric force of the ankle extensors and flexors was reduced in the model of DNG by 30% and 20%, respectively. The muscle force was calculated using static optimization, and peak forces were compared among groups (flexors and extensors of hip, knee, and ankle; ankle evertors; and hip abductors) using MANOVAs, followed by univariate ANOVAs and Newman-Keuls post-hoc tests (*p* < 0.05).

**Results:**

From the middle to late stance phase, DG showed a lower soleus muscle peak force compared to the CG (*p*=0.024) and the DNG showed lower forces in the gastrocnemius medialis compared to the DG (*p*=0.037). At the terminal swing phase, the semitendinosus and semimembranosus peak forces showed lower values in the DG compared to the CG and DNG. At the late stance, the DNG showed a higher peak force in the biceps short head, semimembranosus, and semitendinosus compared to the CG and DG.

**Conclusion:**

Peak forces of ankle (flexors, extensors, and evertors), knee (flexors and extensors), and hip abductors distinguished DNG from DG, and both of those from CG. Both diabetic groups showed alterations in the force production of the ankle extensors with reductions in the forces of soleus (DG) and gastrocnemius medialis (DNG) seen in both diabetic groups, but only DNG showed an increase in the hamstrings (knee flexor) at push-off. A therapeutic approach focused on preserving the functionality of the knee muscles is a promising strategy, even if the ankle dorsiflexors and plantarflexors are included in the resistance training.

**Electronic supplementary material:**

The online version of this article (10.1186/s12984-017-0327-x) contains supplementary material, which is available to authorized users.

## Background

Walking is one of the most automated motor activities in human behavior, with a stereotyped and predictable biomechanical pattern that results from a harmonic coordination between the actions of the neural and musculoskeletal systems. Its regularity and well-described biomechanical patterns allow for objective criteria distinguishing between normal and pathological patterns to be established and, therefore, guide a more focused therapeutic approach aimed at maintaining individuals’ autonomy in daily living activities.

The progression of diabetes mellitus is chronically accompanied by sensory and motor neuropathies, with a marked decrease in somatosensory inputs [1]. Knowledge on how the neuromuscular system of a diabetic neuropathic individual reacts and adapts during gait or functional activities due to his/her motor and functional losses is still a matter of discussion in the literature [2–4]**.** Loss of ankle maximal muscle strength [2, 3, 5, 6] and alterations in distal range of motion (ROM) due to increased stiffness of connective tissue [7, 8], unequal and irregular collagen distribution, increased collagen enzymatic cross-links, and reduced tendon fiber density and diameter [9] have also been well documented in this population. The reduced ankle force and ROM impair the ability of the ankle extensor muscles to push off and generate plantarflexor moments or power during gait terminal stance (70–100% of normalized stance) [10].

Based on published results of net joint moments and joint kinematics in this population [10–13], it is suggested that diabetic individuals pull their leg forward using hip flexor muscles (hip strategy), rather than pushing the leg and superincumbent body weight forward with plantarflexor muscles (ankle strategy) that are dysfunctional. However, net joint moment analysis is insufficient to understand and differentiate individual muscle contributions to the movement and, thus, to establish causal relationships between muscle actions and deteriorated task performance [14]. Furthermore, the investigation of individual muscle forces, such as of the hip flexors and extensors, suffers experimental limitations related mainly to the difficult access to deep muscles and the invasive nature of needle EMG measurements [15].

Complementary to the traditionally reported biomechanical measurements in diabetic individuals, computational simulation studies were developed to address this aforementioned difficulty [16–18]. Computational modeling is used to investigate the role of individual muscles in the generation of joint moments and powers in walking [19], and could be helpful in elucidating possible changes in muscle force distribution that lead to the typical deteriorated gait pattern observed in diabetic patients [20]. In addition, computational studies allow for the determination of whether muscle force changes arise in diabetics prior to the onset of neuropathy or only occur in the presence of neuropathy. To the best of the authors’ knowledge, no studies have estimated lower limb individual muscle forces in diabetic individuals so far.

Understanding both how diabetic patients alter their muscle force production while walking to compensate for the distal deficits (ankle joint complex) and the relative contribution of individual muscles to the force-generating task could guide a more precise rehabilitation approach towards improving the compensations necessary for retaining functional independence in spite of progressive neuromotor distal deficits. Traditionally, routine therapies have focused on muscles in close proximity to the ankle and hip joints, but these therapies lack a firm biomechanical basis. The best approach for rehabilitating the functionality of these patients is still a matter of debate in the literature. For example, increasing distal and/or proximal muscle strength [21], changing the intervention paradigm to focus on the knee [13], and improving connective tissue extensibility [22] or stiffness [23] are all possible rehabilitation strategies. Patients could be trained to use their hip and/or knee muscles more efficiently to compensate for the decreased functionality at the ankle if a better understanding was obtained of how the muscles produce forces and distribute their efforts along the gait cycle while dealing with neural and muscular impairments due to the neuropathy.

The aim of this study was to estimate the distribution patterns of lower-limb muscle forces during walking in diabetic and diabetic neuropathic patients, and to compare those patterns with the ones of non-diabetic individuals using a musculoskeletal model and the static optimization technique. It was expected that (i) muscles crossing proximal joints of diabetic individuals, such as the iliopsoas and rectus femoris (hip flexors), would alter their activation, compensating for a distal reduction of force (ankle dorsiflexors and plantarflexors), and that (ii) a substantial change in the force distribution of proximal and distal muscles would be observed in diabetic neuropathic individuals.

## Methods

### Participants

Thirty adults below 65 years of age were evaluated and divided into a control group of non-diabetic participants (CG, *n* = 10), a diabetic group without the presence of polyneuropathy (DG, *n* = 10), and a diabetic group with a clinical diagnosis of diabetic neuropathy (DNG, *n* = 10). The exclusion criteria for the three groups included partial or total lower limb amputation, presence of a Charcot arthropathy (or any other major orthopedic impairment confirmed by x-ray), neuromuscular disease, central or peripheral neurological disease not caused by diabetes mellitus, severe retinopathy or nephropathy, plantar ulcer at the time of evaluation, and the inability to walk independently without pain or the use of an assistive device. Diabetic participants were recruited from the National Association of Diabetes. Inclusion in the DNG was decided according to the following criteria: type 2 diabetes diagnosed for at least five years, score greater than 3 out of a maximum of 13 points in the Michigan Neuropathy Screening Instrument (MNSI) questionnaire, and a score greater than 4 points on a 10-point scale for physical assessment. Both instruments for performing these measurements were previously validated for the identification of diabetic neuropathy symptoms [24]. All procedures were approved by the Research Ethics Committee of the Faculty of Medicine, University of Sao Paulo (Protocol No. 239/13), and all participants signed an informed consent form prior to participation.

The three groups were similar in age, height, mass, and body mass index. The DNG presented longer periods of diabetes mellitus diagnoses and higher scores in the MNSI-questionnaire compared to the DG, as well as tactile and vibratory perception impairments (Table 1).

**Table 1:** Demographic and clinical characteristics of each group [mean (standard deviation)]

	CG (*n* = 10)	DG (*n* = 10)	DNG (*n* = 10)	*P*-value
Age (years)	49.7 (4.7)	54.5 (7.4)	56.7 (7.3)	0.096^*^
Sex (n° men)	5	8	6	0.579^†^
Height (m)	1.69 (0.07)	1.68 (0.1)	1.63 (0.11)	0.295^*^
Body mass (kg)	69.4 (7.8)	75.1 (10.5)	71.7 (11)	0.462^*^
Body mass index (kg/m^2^)	23.9 (2.2)	26.0 (5.2)	27.0 (3.4)	0.163^*^
Diabetes duration (years)	--	5.8 (3.4)	16.6 (6.2)^‡^	**<0.001** ^*^
MNSI-questionnaire (median)	--	2	8^‡^	**0.001** ^*^
Tactile perception^#^	0	0	6^‡^	**0.003** ^†^
Halux vibratory perception^&^	0	0	8^‡^	**0.003** ^†^

^*^ANOVA one way. ^†^Chi-square. ^‡^represent the statistically different group. Bold (*p* < 0.05). ^#^n° of individuals with tactile perception compromised in more than 3 foot areas. ^&^n° of individuals without vibratory perception in, at least, one foot

### Data acquisition

Six infrared cameras with a motion capture system were used (OptiTrack FLEX: V100; Natural Point, Corvallis, OR). A full volume test for the accuracy of the OptiTrack System was performed, as suggested by Chiari et al. (2005) [25]. The results of the static and dynamic tests showed an error lower than 1 mm within a volume of 1.6 m x 1.2 m x 2.4 m (L x W x H), similar to other commercial systems usually used in biomechanics [26]. Body motion was tracked by attaching reflective markers (18 mm diameter) bilaterally at the volunteer’s feet, legs, thighs, and trunk, following the Cleveland Clinic marker set [27]. In addition, rigid clusters with four markers each were fixed laterally on the thighs and shanks. The evaluated limb was randomized among participants by a simple draw. For the ground reaction force simultaneous acquisition, a triaxial force plate (OR6-7-1000; AMTI, Watertown, MA) embedded in the center of a walkway was used (12-bits A/D converter, AMTI, DT 3002; sampling rate 100 Hz).

The participants were instructed to walk barefoot at a self-selected speed. To avoid major differences in walking speed among participants and groups, the gait cadence was monitored to ensure it remained between 96 and 120 steps per minute using a metronome in a mute mode. The cadence of all the participants remained within this range throughout the trials, so no intervention was required for cadence adjustment. Trials were recorded after a habituation period in the laboratory environment. The habituation consisted of a five minutes period when each participant was instructed to walk through the laboratory as he/she usually does on a daily basis. After the habituation period, six trials were acquired and three valid ones were chosen for statistical purposes. The valid trials were characterized by the absence of movement artifacts from the body markers in the model reconstruction, perfect synchronization between kinematics and ground reaction forces, as well as the presence of one complete gait cycle. Data acquisition was always performed by the same physical therapist to avoid possible differences in the placement of body markers.

### Musculoskeletal model and muscle force estimation

The kinetic and kinematic data were processed in the Visual3D software (C-Motion, Kingston, ON, Canada) to generate inverse kinematics data and subject-specific scaling factors using a model specifically developed to allow for data exportation for use in OpenSim. Computed joint moments and kinematics of the control group showed an excellent adherence to the literature data [4]. In OpenSim v.3.2 [28], after importing the data from Visual3D, a generic model of the lower extremities and trunk, with 23 degrees of freedom and 92 Hill-type musculoskeletal actuators [28] (Gait2392), was scaled to each individual’s height, body mass, and segment geometry.

In order to approximate the computational model of patients with diabetic neuropathy, 30% of the maximal isometric force of the ankle extensor muscles (gastrocnemius medialis and lateralis, soleus) and 20% of the ankle flexor muscle (tibialis anterior) was deducted in the musculoskeletal models of the DNG, according to average values reported in the literature [2, 29]. There is little information available in the literature about the muscle properties of diabetic individuals other than muscle strength except for murine models [30]. Thus, the maximal isometric force was the only parameter that was changed to approximate the musculoskeletal model to the DNG group in an effort to adapt the model according to the current state of knowledge. Although other musculoskeletal parameters have been shown to affect the results for walking [31], the lack of information on these specific parameters in the diabetic population prevented their implementation on the musculoskeletal model.

The static optimization algorithm implemented in OpenSim was used to estimate the muscle forces [32] from bilateral kinematics, ipsilateral ground reaction forces, and the individuals’ scaled musculoskeletal models. The algorithm searches muscle forces that minimize the sum of squared activations and that satisfy the equations of motion, physiological upper and lower bounds on muscle activations, and constraints imposed by the force-velocity and force-length relationships, assuming that the tendons are inextensible. This algorithm is computationally efficient, does not require multiple numerical integrations, and generates solutions similar to those for dynamic optimizations for gait [33]. The cost function used is defined as follows:

$$ J={\Sigma}_{i=1}^m{a}_i^2, $$

where *m* is the number of muscle units considered in the model and *a*_*i*_ is the activation of the *i* ^*th*^ muscle*.*

The adopted quadratic optimization cost associated with static optimization was also shown to produce the best agreement between electromyography and muscle activation patterns and estimates of hip contact forces [34, 35]. Steele et al. [36] used a weighted version of this cost function to investigate crouch gait, which involves neuromuscular disorders generally more severe than diabetes; thus, this function could also be a suitable criteria for diabetic individuals.

### Data Analysis

Kinematic and kinetic data were filtered using a fourth-order zero-lag low-pass Butterworth filter with cut off frequencies of 6 Hz and 20 Hz, respectively.

The following were calculated for the whole gait cycle: hip, knee, and ankle joint angular displacements from the inverse kinematics (Additional file 1: Figure S1), as a preliminary stage to the muscle force estimation; net joint moments (Additional file 1: Figure S2), and peak muscle forces calculus. The time series of each physical quantity was normalized in time from 0 (heel contact on the force plate) to 100% of the gait cycle (next heel contact of the same limb). Muscle forces and net joint moments were also normalized by body weight (BW) and by the product of BW and height, respectively.

The variables used to describe and compare the three groups (CG, DG, and DNG) were the peak muscle forces at the intervals in the gait cycle in which the muscle activations were fundamental to the task [37]. The muscles and respective intervals studied were as follows:

- Hip flexors: psoas (40–60%), iliacus (40–60%);
- Hip extensors: 3 portions of gluteus maximus (0–35%);
- Hip abductors: 3 portions of gluteus medium (0–35% and 35–60%);
- Hip extensors/knee-flexors: biceps femoris short head (40–60% and 80–100%), biceps femoris long head (40–60% and 80–100%), semitendinosus (40–60% and 80–100%), semimembranosus (40–60% and 80–100%);
- Knee extensors: 4 portions of quadriceps femoris (vastus medialis, vastus intermedius, vastus lateralis, rectus femoris) (0–35%);
- Ankle flexors: tibialis anterior (0–30% and 40–80%) and extensor hallucis longus (0–30% and 40–80%);
- Ankle extensors: gastrocnemius medialis (30–50%), gastrocnemius lateralis (30–50%), soleus (30–50%), flexor hallucis longus (0–35% and 35–60%);
- Ankle evertors: peroneus longus (0–35% and 35–60%), peroneus brevis (0–35% and 35–60%).

Two types of analyses were performed considering the large amount of data: a comparative statistical multivariate analysis to identify changes in the muscle force estimation among groups, and a qualitative analysis, which make it possible to qualitatively observe the differences among time series of the studied groups to recognize and discuss potential pattern changes, possibly not identified by the statistical analysis [38]. We consider a change greater than ten percent as a notable increase or decrease, and the only variables discussed qualitatively were iliopsoas and iliacus peak forces and the instant of ankle extensors activation.

To evaluate the muscle force estimation, the force patterns were analyzed at selected intervals and compared amongst the three groups by eight MANOVAs, one for each of the following muscle groups: hip flexors, hip extensors, hip extensors-knee flexors, knee extensors, hip adductors, ankle flexors, ankle extensors, and ankle evertors. If significance was achieved (*p* < 0.05), MANOVAs were followed by univariate ANOVAs, which, in turn, were followed by Newman-Keuls post hoc tests. To avoid multiple comparisons unnecessary and thus inflation of type I error, ANOVAs and post hoc comparisons were only additionally performed if the MANOVAs were significant flexor and extensor hallucis longus muscles were not included in the MANOVA analyses because of the almost negligible force production of these muscles, compared to all other ankle muscles. In addition to that, considering the simple and non-segmented foot model in the gait 2392 model, it was advisable not to deepen and further extrapolate the results of hallux muscles. The calculated force values of these muscles were presented in the supplementary material, together with their ANOVA analyses.

The results of each comparison were reported together with Cohen’s d effect size where in a small effect is considered between 0.2–0.4; intermediate effect between 0.5–0.7, and large effect from 0.8 up to 1.0 [39]. All of these comparisons were performed to map the force distribution patterns within patient groups and identify the direction of change among the groups. For all variables, normal distribution (Shapiro Wilk test) and homoscedasticity (Levene Test) were achieved. For the sample size evaluated, a power (1-beta) of 0.74 was achieved for an F test designed for 3 groups (*n*=30), an alpha of 5%, and a mean effect size of 0.56 considering the significant ANOVAs.

## Results

There was no significant difference in gait speed among the groups (CG: 1.18 ± 0.10 m/s; DG: 1.12 ± 0.09 m/s; DNG: 1.12 ± 0.13 m/s; *p* = 0.056 [ANOVA]).

### Qualitative analysis

**Fig. 1:**
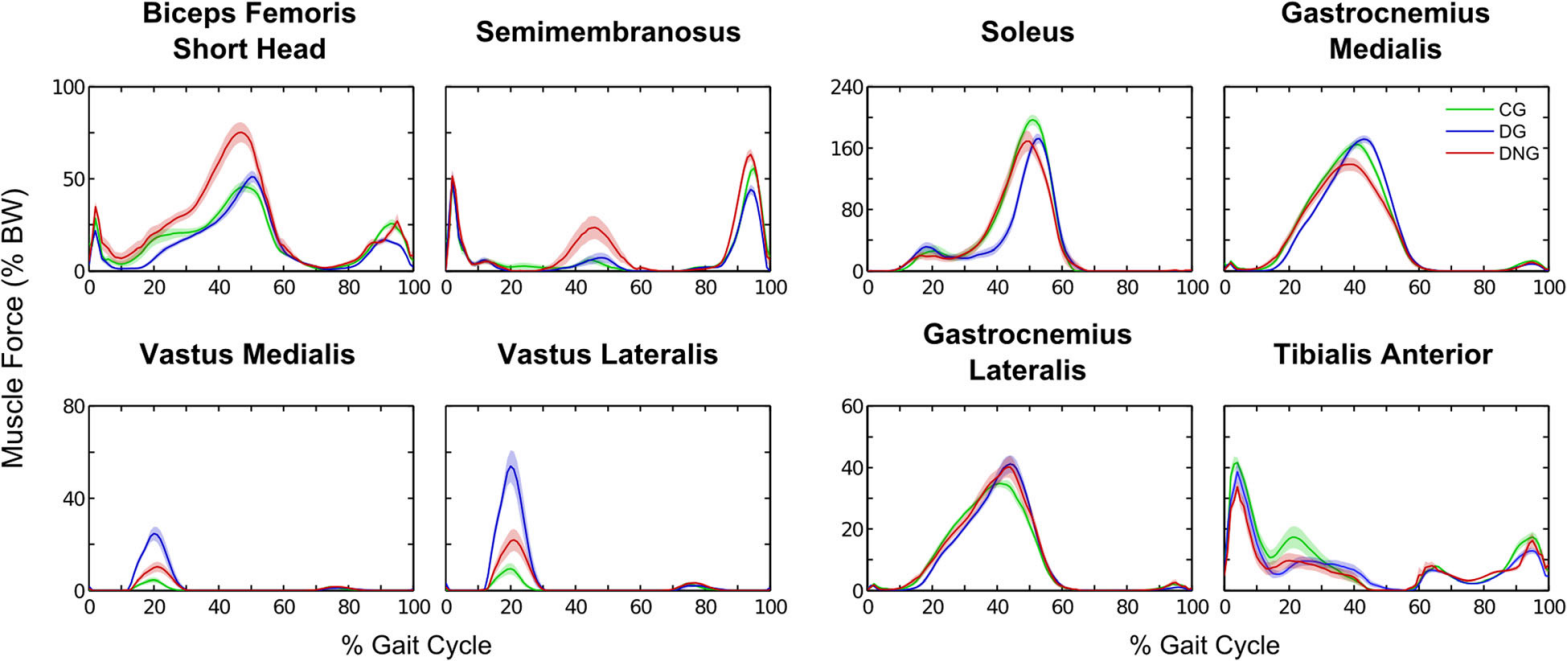
Mean (± 1 standard error) of the force time series for the knee flexors and hip extensor muscles (biceps short head and semimembranosus), knee extensors muscles (vastus medialis and vastus lateralis), ankle extensor muscles (soleus, gastrocnemius medialis, and lateralis), and ankle flexor muscle (tibialis anterior) in the control group (CG - green), diabetic group (DG – blue), and diabetic neuropathy group (DNG – red), during the gait cycle

**Fig. 2:**
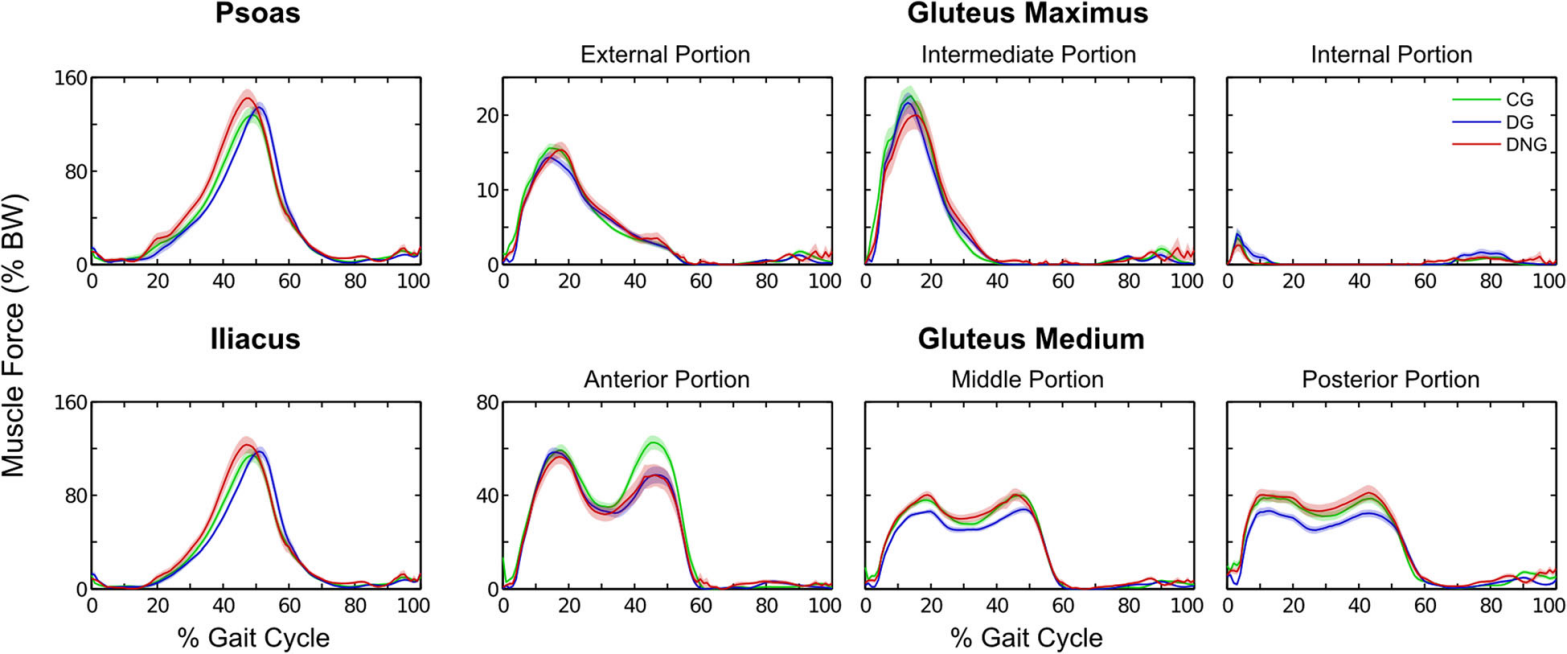
Mean (± 1 standard error) of the force time series for the hip flexors (psoas and iliacus), hip extensors (external portions and intermediate gluteus maximus), and hip abductor muscles (anterior and middle portions of gluteus medius) in the control group (CG - green), diabetic group (DG - blue), and diabetic neuropathy group (DNG - red), during the gait cycle

A qualitative analysis of the muscle force patterns suggests that both diabetic groups present difficulty in generating force in some ankle extensor muscles, where soleus peak forces were lower compared to healthy individuals in the push-off phase. Particularly, DG individuals presented a late activation of ankle extensors compared to the other groups (Fig. 1). The DNG showed a gastrocnemius medialis peak force reduction in the push-off phase, but the diabetic groups were not different between them in the gastrocnemius lateralis peak force (Fig. 1). The DNG increased force production of hip and knee flexors (psoas, iliacus, and hamstrings) in the push-off phase (Figs 1 and 2), and the DG did not change the force generation of these muscles at this phase. Both diabetic groups exhibited smaller force of the anterior portion of gluteus medium in the second half of the stance compared to the CG, and the DG also showed a smaller force of the posterior portion of this muscle compared to the CG in this phase, revealing that the force production of the gluteus medium is impaired in the DG (Fig. 2). Patients of the DG also presented an increased peak force of the vastus lateralis and medialis in the initial stance (0–35%), which was consistent with their larger knee extensor moment (Additional file 1: Figures S2 and S3).

### Quantitative analysis

#### Hip flexor and extensor muscles

The results of the MANOVA showed no difference among the groups for the hip flexors and extensors in the selected gait cycle intervals. The univariate ANOVAs also did not show significant differences among the groups in any muscle or phase (Table 2, Fig. 2).

**Table 2:** Hip muscle’s peak force values (mean ± standard deviation) normalized by body weight (BW) of diabetic neuropathy (DNG), diabetic (DG) and control (CG) groups during the gait cycle

Muscles (% Gait Cycle)		Force Peak (% BW)					
		CG^1^ (*n*=10)	DG^2^ (*n*=10)	DNG^3^ (*n*=12)	p Wilks’ Lambda^*^	dCohen	*p* ^†^
Hip Flexors	Psoas (40 – 60%)	134.5 ±36.8^a^	140.7 ±26.0^b^	156.1 ±30.6^a,b^	0.129^*^	__	__
	Iliacus (40 – 60%)	120.1 ±32.0	124.6 ±24.2	138.7 ±29.8		__	__
Hip Extensors	Gluteus Maximus– external portion (0 – 35%)	16.9 ±3.6	16.3 ± 1.8	16.3 ±4.61	0.669^*^	__	__
	Gluteus Maximus– intermediate portion (0 – 35%)	24.8 ±8.2	23.1 ±6.6	22.1 ±7.9		__	__
	Gluteus Maximus– internal portion (0 – 35%)	4.6 ±5.9	5.2 ±3.4	3.79 ±5.5		__	__
Hip Abductors	Gluteus Medium– anterior portion (0 – 35%)	63.5 ±12.5	61.6 ±8.7	59.6 ±15.1	**0.001** ^*^	(1x2) -0.176 (1x3) -0.281 (2x3) -0.162	0.335^†^
	Gluteus Medium– anterior portion (35 – 60%)	64.5 ±15.9^a,b^	51.7 ±18.5^a^	53.0 ±21.8^b^		(1x2) -0.742 (1x3) -0.603 (2x3) 0.064	**0.028** ^†^
	Gluteus Medium– middle portion (0 – 35%)	41.3 ±8.1^a^	34.8 ±4.2^a,b^	39.6 ±9.7^b^		(1x2) -1.007 (1x3) -0.190 (2x3) 0.642	**0.005** ^†^
	Gluteus Medium– middle portion (35 – 60%)	40.9 ± 7.2^a^	34.7 ±5.9^a,b^	41.7 ±7.4^b^		(1x2) -0.942 (1x3) 0.110 (2x3) 1.046	**0.013** ^†^
	Gluteus Medium– posterior portion (0 – 35%)	45.1 ±9.2^a^	37.6 ±6.0^a,b^	42.3 ±12.5^b^		(1x2) -0.966 (1x3) -0.255 (2x3) 0.479	**0.003** ^†^
	Gluteus Medium– posterior portion (35 – 60%)	38.7 ±8.4	33.8 ±8.6^a^	47.9 ±11.8^a^		(1x2) -0.576 (1x3) 0.898 (2x3) 1.366	**0.012** ^†^

^*^MANOVA, ^†^Univariate ANOVA**,** ^a,b^Post-hoc Newman-Keuls ^(a, b^showed the significantly different pair of values between the groups, *p*<0.05 adopted), 1 represent CG, 2 represent DG and 3 represent DNG. Bold (*p*<0.05)

#### Hip abductor muscles

The hip abductor muscles differed among the groups in the MANOVA analysis, with the DG showing lower forces compared to the CG and DNG. According to the univariate ANOVAs, at the initial stance (0–35% gait cycle), the DG showed lower gluteus medium force (posterior and middle portions) compared to the CG (large effect) and DNG (large effect). In the late stance (35–60% gait cycle), the DG also showed lower peak forces in the three portions of the gluteus medium compared to the CG (anterior and posterior portions with intermediate effect sizes and middle with large), and in the middle portion of the gluteus medium compared to the DNG (intermediate effect size) (Table 2, Fig. 2).

#### Hip extensor- knee flexor muscles

Hip extensor- knee flexor muscles showed differences among the groups in the MANOVA analysis. At the end of the swing phase (80–100% gait cycle), the semitendinosus and semimembranosus peak forces showed lower values in the DG compared to the CG and DNG (both comparisons with large effect sizes) (Table 3, Fig. 1). At the late stance (40–60% gait cycle), the DNG showed a higher peak force in the biceps short head, semimembranosus, and semitendinosus with a large effect compared to the CG and DG (Table 3, Fig. 1).

**Table 3:** Hip extensors-knee flexors and knee extensors muscle’s peak force values (mean ± standard deviation) normalized by body weight (BW) of diabetic neuropathy (DNG), diabetic (DG) and control (CG) groups during the gait cycle

	Muscles (% Gait Cycle)	Force Peak (% BW)					
		CG^1^ (*n*=10)	DG^2^ (*n*=10)	DNG^3^ (*n*=12)	p Wilks’ Lambda^*^	dCohen	*p* ^†^
Hip Extensors- Knee Flexors	Biceps Fem. Short Head (40 – 60%)	51.1 ±15.5^a^	57.8 ±18.7^b^	80.4 ±25.5^a,b^	**<0.001** ^*^	(1x2) 0.390 (1x3) 1.389 (2x3) 1.011	**<0.001** ^†^
	Biceps Fem. Short Head (80 – 100%)	32.0 ±12.6^a^	19.6 ±8.3^a^	27.3 ±5.6 ^a^		(1x2) 1.162 (1x3) 0.482 (2x3) 1.088	**<0.001** ^†^
	Biceps Fem. Long Head (40 – 60%)	0.3 ±0.8	0.7 ±2.5	3.45±9.0		(1x2) 0.216 (1x3) 0.493 (2x3) 0.416	0.072^†^
	Biceps Fem. Long Head (80 – 100%)	20.4 ±6.1	15.6 ±5.6	15.1 ±11.3		(1x2) -0.820 (1x3) -0.584 (2x3) -0.056	0.060^†^
	Semitendinosus (40 – 60%)	1.42 ±2.4^a^	0.17±0.5^b^	6.1 ±8.2^a,b^		(1x2) -0.721 (1x3) 0.775 (2x3)1.021	**<0.001** ^†^
	Semitendinosus (80 – 100%)	5.6 ±1.9^a^	4.3 ±1.5^a,b^	6.0 ±1.6^b^		(1x2) -0.759 (1x3) 0.228 (2x3) 1.096	**<0.001** ^†^
	Semimembranosus (40 – 60%)	6.4±10.0^a^	7.2±12.1^b^	29.4±31.3^a,b^		(1x2) 0.072 (1x3) 0.990 (2x3) 0.936	**<0.001** ^†^
	Semimembranosus (80 – 100%)	62.0 ±13.6^a^	45.1 ±13.7^a,b^	68.5 ±9.7^b^		(1x2) 1.238 (1x3) 0.550 (2x3) 1.971	**<0.001** ^†^
Knee Extensors	Vastus Lateralis (0 – 35%)	13.8 ±11.9^a^	29.7 ±21.5^a,b^	19.0 ±19.7^b^	**0.034** ^*^	(1x2) 0.915 (1x3) 0.320 (2x3) -0.519	**0.004** ^†^
	Vastus Intermedius (0 – 35%)	8.8 ± 7.8	17.8 ±13.2	11.8 ±11.7		(1x2) 0.830 (1x3) 0.302 (2x3) -0.481	0.293^†^
	Vastus Medialis (0 – 35%)	6.6 ± 5.5^a^	13.44 ±9.3^a,b^	8.7 ±9.0^b^		(1x2) 0.895 (1x3) 0.282 (2x3) -0.518	**0.004** ^†^
	Rectus Femoris (0 – 35%)	44.5 ± 17.7	51.93 ±21.5	52.9 ±22.1		(1x2) 0.377 (1x3) 0.420 (2x3) 0.044	0.941^†^

^*^MANOVA, ^†^Univariate ANOVA, ^a,b^Post-hoc Newman-Keuls ^(a,b^showed the significantly different pair of values between the groups, *p*<0.05 adopted), 1 represent CG, 2 represent DG and 3 represent DNG. Bold (*p*<0.05)

#### Knee extensor muscles

The MANOVA output of the knee extensor muscles revealed differences among the groups. Univariate ANOVAs showed that in the early stance (0–35% gait cycle), the DG exhibited higher peak forces in the vastus medialis and lateralis compared to the CG and DNG (large and intermediate effect sizes, respectively) (Table 3).

#### Ankle flexor muscles

The MANOVA results did not show differences among the groups, and according to univariate ANOVAs, there were no differences in the tibialis anterior in any phase interval (Table 4, Fig. 1). The DNG exhibited a higher extensor hallucis longus peak force compared to the DG and CG (intermediate and small effect sizes, respectively) (Additional file 1: Table S3, Figure S3).

**Table 4:** Ankle muscle’s peak force values (mean ± standard deviation) normalized by body weight (BW) of diabetic neuropathy (DNG), diabetic (DG) and control (CG) groups during the gait cycle

	Muscles (% Gait Cycle)	Force Peak (% BW)					
		CG^1^ (*n*=10)	DG^2^ (*n*=10)	DNG^3^ (*n*=12)	p Wilks’ Lambda^*^	dCohen	*p* ^†^
Ankle Flexors	Tibialis Anterior (0 – 30%)	58.5±16.5	62.7±27.4	66.6±23.4	0.059	__	__
	Tibialis Anterior (40 – 80%)	10.2 ±1.5	11.9 ±6.1	9.1 ±1.1		__	__
Ankle Extensors	Gastrocnemius Medialis (30– 50%)	173.6 ±29.3	183.0 ±30.5^a^	158.7 ±37.6^a^	**<0.001** ^*^	(1x2) 0.314 (1x3) -0.442 (2x3) -0.710	**0.037** ^†^
	Gastrocnemius Lateralis (30 – 50%)	38.0 ±8.6^a^	42.7 ±15.6^b^	44.8 ±17.7^a,b^		(1x2) 0.373 (1x3) 0.489 (2x3) 0.126	**0.021** ^†^
	Soleus (0 – 30%)	35.2 ±37.3	37.1 ±31.1	29.0 ±26.2		(1x2) 0.055 (1x3) -0.192 (2x3) -0.282	0.666^†^
	Soleus (40 – 60%)	213.0 ±39.9^a^	183.1 ±29.3^a^	195.8 ±48.2		(1x2) -0.854 (1x3) -0.389 (2x3) 0.318	**0.024** ^†^
Ankle Evertors	Peroneus Longus (0 – 35%)	11.9 ±8.3^a^	26.6 ±18.4^a,b^	17.3 ±12.4^b^	**<0.001** ^*^	(1x2) 1.030 (1x3) 0.512 (2x3) -0.593	**<0.001** ^†^
	Peroneus Longus (35 – 60%)	25.1 ±19.5^a^	31.5 ±19.1^b^	64.2 ±35.9^a,b^		(1x2) 0.332 (1x3) 1.353 (2x3) 1.137	**<0.001** ^†^
	Peroneus Brevis (0 – 35%)	4.0 ±2.6^a^	7.9 ±5.4^a,b^	5.3 ±3.5^b^		(1x2) 0.920 (1x3) 0.422 (2x3) -0.571	**0.003** ^†^
	Peroneus Brevis (35 – 60%)	4.9 ±3.5^a^	7.2 ±4.6^b^	12.4 ±7.8^a,b^		(1x2) 0.563 (1x3) 1.241 (2x3) 0.812	**<0.001** ^†^

^*^MANOVA, ^†^Univariate ANOVA, ^a,b^Post-hoc Newman-Keuls ^(a,b^showed the significantly different pair of values between groups, *p*<0.05 adopted), 1 represent CG, 2 represent DG and 3 represent DNG. Bold (*p*<0.05)

#### Ankle extensor muscles

The MANOVA results showed differences among the groups and the main differences in the univariate ANOVAs were observed from the middle to late stance phase (30–50% gait cycle), where the DG showed a lower soleus muscle peak force compared to the CG (large effect size) (Table 4, Fig. 1), with a consequent lower peak ankle joint moment compared to the CG and DNG (Additional file 1: Table S2, Figure S2). At the same gait interval, the DNG showed lower forces in the gastrocnemius medialis compared to the DG (intermediate effect), but higher in the gastrocnemius lateralis compared to the DG and CG (small and intermediate effect sizes, respectively) (Table 4, Fig. 1).

#### Ankle evertor muscles

The MANOVA output of the evertor muscles (peroneus longus and brevis) revealed differences among the groups. Univariate ANOVAs showed that at an early stance (0–35% gait cycle), the DG showed higher peak forces for both muscles. At a late stance (35–60% gait cycle), the DNG revealed higher peak forces for both muscles compared to the CG and DNG (all large effect sizes) (Table 4, Fig. 1).

## Discussion

The focus of this study was to investigate how diabetic individuals with and without neuropathy alter their muscle activation and force production to compensate for the greater involvement of distal musculoskeletal structures consequent to the disease. The main findings partially confirmed the hypothesis, as there was observed ankle plantarflexor force reduction in the DG and DNG, but surprisingly, the proximal muscles (iliopsoas, iliacus, and rectus femoris) did not show a significant increase in their peak force. There was not an accentuated change in the force distribution of the proximal and distal muscles between the controls and the diabetic patients, and from those to the diabetic neuropathic patients, as was hypothesized. Both diabetic groups showed alterations in the force production of the ankle extensors, with reductions in the forces of soleus (DG) and gastrocnemius medialis (DNG), but only the DNG had increased forces of the hamstrings (knee flexor) at push-off. The DG presented a greater reduction in the force production of gluteus medium, and this might suggest a consequence for the latero-lateral stabilization of the pelvis.

Ankle extensor muscles perform distinct mechanical functions over different joints and distribute power in particular ways [16]. Thus, their individual alterations in the DG and DNG groups lead to different consequences in their proximal joints. The DG and DNG used more knee muscles but with different patterns of force distribution of the biarticular knee flexor muscles (hamstrings). The DNG showed an increase in the semitendinosus and semimembranosus peak forces in the transition between midstance and push off; this strategy may have enhanced the chance of a successful toe clearance by increasing the knee flexion in the swing phase. The DG patients showed a reduction in the force of the same muscles at the end of swing phase, which can potentially impair the deceleration of the limb at the end of the gait cycle. The concurrent higher forces of the hip antagonists (biarticular hamstrings and uniarticular psoas) at the transition phase between stance and swing can explain the lower net hip flexion moment in the DNG.

Lower maximal isometric force of the tibialis anterior has been described in diabetic neuropathic individuals [2, 3, 5], and the DNG model in this study was modified to approximate this alteration by reducing 20% of its maximum isometric force. Contrary to expectations, there was no observable reduction in the tibialis anterior peak force during walking. Possibly, the DNG patients had to increase the muscle activation to guarantee a muscle force production similar to the CG in a condition of lower maximum isometric force of the ankle flexor. This action would result in a potential increase in muscle-specific metabolic cost in DNG individuals to perform the same motor task. In fact, it has been shown that the DNG patients had a higher metabolic cost despite their reduced concentric lower limb joint work compared with the controls at matched speeds, and there were several kinematic gait alterations with implications for joint kinetics [40].

The same reasoning used for the absence of significant alterations in the tibialis anterior could be applied to all ankle extensors (gastrocnemius medialis and lateralis, soleus) whose maximal isometric forces were reduced by 30% in the model. To maintain a similar force pattern of these muscles, it is also plausible that the DNG patients had to increase the corresponding muscle activations. Thus, a focus on therapeutic strategies directed to muscle efficiency could help these patients maintain a more functional gait. However, both assumptions for ankle extensors and flexors need further EMG confirmation.

DNG patients exhibited a 14% and 10% increase in psoas muscle forces compared to the DG and CG, respectively. Although this increase was not statistically significant, the psoas muscle is a powerful monoarticular hip flexor, and the DNG presented a lower soleus muscle force – a monoarticular ankle extensor, at the transition between stance and swing (40–70% of gait cycle) compared to the DG and CG. The individual contributions of the distal and proximal muscles at the push-off phase of the DNG might suggest the existence of the hip strategy at the individual-muscle level, particularly for the monoarticular muscles of the ankle and hip, although there was no observed reduction in the net ankle extensor moment or increase in the net hip flexor moment, which characterize the so-called hip strategy, as previously reported [10].

Regarding the progressive neuromuscular damage due to diabetic neuropathy and the force alterations of the hamstrings in the DNG patients, a resistance training program for the proximal muscles related to the knee joint could be considered in a rehabilitation routine for diabetic patients. Other potential inclusions in rehabilitation protocols consist of gait retraining and practicing functional exercises focusing on the activation of the hamstring muscles. Considering the progression of the disease and the difficulty in maintaining distal muscle force, a therapeutic approach focusing on the functionality preservation of the knee muscles is a promising strategy, even if the ankle dorsiflexors and plantarflexors are also included in the resistance training. Training motor tasks that challenge the motion control of ankle and knee would be helpful in stimulating the neuromuscular system to create new motor solutions. Tasks such as walking, changing directions, cutting maneuvers, traversing obstacles, and standing on uneven surfaces could help incorporate gains into daily functional activities.

Interesting changes were observed in the force production of muscles that contribute to the movement and stability in the frontal plane. The DNG individuals increased the peroneous force (longus and brevis) at midstance and push-off, whereas the DG increased these muscle forces in the early stance. In the early stance, the peroneal muscles act to limit excessive rear foot inversion; at midstance they act to decelerate subtalar pronation and assist in the stabilization of the midfoot joints, and at push-off, these muscles contribute to plantar flex the hallux. The DG may have recruited the evertor muscles to favor foot alignment in the early stance, and the DNG recruited the same muscles to assist the sagittal propulsion accomplished by the foot and ankle complex, probably due to the important reduction in the ankle extensors in the same phase.

Considering the frontal plane alterations, the DG patients also showed a reduced gluteus medium force at midstance that could lead to an alteration in the latero-lateral stabilization of the pelvis in the single leg stance. Sawacha et al. [12] also observed an alteration in gluteus medium action during walking, represented by a delay in its activation peak in diabetic non-neuropathic individuals during terminal swing. They discuss that the major muscle groups are active in the periods of deceleration and acceleration of the legs (at or around both heel strike and toe-off), when body weight is transferred from one limb to the other. They found that these are the gait phases where either neuropathic or non-neuropathic individuals differed significantly from healthy, which corroborates with our results for gluteus medium and some other muscles, as gastrocnemius medialis and lateralis, or vastus lateralis. Although the optimal choice of muscle targets for inclusion in physiotherapy routines remains contentious, a functional strengthening program of the gluteus medium in DG patients could favor latero-lateral pelvic stabilization during the gait cycle.

Surprisingly, the DG patients did not present a force production pattern that was intermediate between controls and diabetic individuals with neuropathy. They showed a particular force distribution pattern, different from the other two groups. Sawacha et al. [41] used a cluster analysis to verify if three-dimensional gait data would be able to distinguish neuropathic, non-neuropathic diabetic patients and healthy individuals. They found two clusters which contained both neuropathic and non-neuropathic diabetic patients, showing that there is no single gait pattern for each pathologic population. Williams et al. [13] also observed an alteration in gait biomechanics of non-neuropathic diabetic patients, which, similarly, could not be recognized as intermediate between healthy controls and neuropathic patients

Diabetic patients present neuromuscular alterations even before clinical evidence of neuropathy [42]. This early neuromuscular impairment was confirmed by the lower conduction velocities of nerves even before the loss of muscle force or appearance of neuropathy symptoms, suggesting that diabetes mellitus progression is concomitant for both neuromuscular and sensory systems and not a late complication of neuropathy [43], as previously assumed in the literature. The skeletal muscles are acutely sensitive to diabetes mellitus prior to neuropathic complications [44, 45], and the altered peak force of the soleus, hamstrings, knee extensors, and gluteus medium observed in DG patients corroborates this thesis. The muscle force redistribution pattern associated with other musculoskeletal dysfunctions in diabetic patients [42–46] could also be responsible for their cautious gait pattern, represented, for instance, by a lower velocity and a longer double stance phase [47]. Therefore, an exercise program including gait retraining for non-neuropathic diabetic patients could assist in maintaining ankle muscle capacity and increase proximal muscle capacity to preserve walking ability, which would guarantee independence as patients deal with their neural and muscular impairments due to the neuropathy.

Some methodological limitations should be considered when interpreting these results. First of all, although the statistical design adopted is robust enough for this type of analyses, study question and database, we might have increased the chance of type I error due to multiple comparisons after MANOVAs*.* As in all model-based analyses in biomechanics, estimations depend on a myriad of model parameters describing musculoskeletal geometry, joint properties, anthropometry, and intrinsic muscle properties, among others [48]. In fact, some Hill-type muscle properties have been shown to affect results for walking [31]. Unfortunately, to date, there are no available data on these musculoskeletal properties for diabetic patients regardless of neuropathy diagnosis, except on maximal isometric forces for neuropathic patients. For this reason, this DNG model could only be adjusted to approximate the gait of neuropathic individuals by changing the maximum isometric force of the distal muscles whose alterations have been extensively described in the literature. Thus, careful interpretation of results is recommended as for any subject-specific model-based study. The non-neuropathic model remained unchanged when compared to the control group model. Any other changes to the musculoskeletal models would be highly speculative and potentially non-representative of all the individuals of a group. Overcoming this limitation would require measuring or estimating the patient-specific properties of each subject, which would be currently unfeasible, or performing a large-scale sensitivity analysis on the hundreds of parameters, which would be impractical.

Additionally, solving the muscle redundancy problem through static optimization] requires the adoption of a cost function [32], which is another potential source of inaccuracies in muscle force estimations in this kind of study. The sum of squared muscle activations adopted as a cost function in the present study is widely used to estimate muscle force distribution in various tasks [32], including pathological gait such as crouch gait [36], and is the standard cost function in the static optimization tool of the OpenSim software. Moreover, it has been shown that this quadratic cost function associated with static optimization has provided muscle force estimations similar to the ones obtained by dynamic optimization [33] as well as good adherence to EMG patterns and best estimates of hip contact forces in walking [34, 35].

Although the use of generic models and cost functions will probably lead to inaccuracies in muscle force estimations, the presented study does, at a minimum, provide trends and directions of changes in muscle force distributions and offers a solid basis for the formulation of hypotheses to be confirmed in upcoming studies. In view of the aforementioned limitations, future studies should focus on the development of representative models of the diabetic population, which in turn will require collecting quantitative, currently unavailable, data on typical musculoskeletal properties of diabetic individuals in different stages of the disease. In addition, further studies should also focus on the determination of an optimal objective function to advance our understanding of diabetic gait strategies.

In summary, the results indicate that muscle forces of the ankle (flexors, extensors, and evertors), knee (flexors and extensors), and hip abductors distinguished the gait pattern of diabetic individuals with neuropathy from diabetic individuals without neuropathy and from the controls. The findings are expected to contribute to more focused therapeutic interventions based on the relative contribution of muscles to the gait patterns adopted by each group. Taking into account that preserving the force capacity of the ankle extensors is critical for the maintenance of normal walking, it is important to intervene in the function of these muscles in diabetic patients with or without neuropathy because of their earlier impairment in the course of the disease. Moreover, it is important to maintain and improve the function of the proximal muscle groups, particularly the hamstrings, in response to the force deficit of the ankle muscles.

## Conclusion

In conclusion, there are four main takeaway points from this study. First, there was no observable progressive worsening in gait patterns from the controls to diabetic patients and from those to the diabetic neuropathic patients. Instead, each diabetic group showed a particular and distinct adaptation pattern in terms of muscle force distribution. Second, both diabetic groups showed alterations in the force production of the ankle extensors, with reductions in the forces of soleus (DG) and gastrocnemius medialis (DNG). Third, diabetic patients compensated for deficiencies in their ankle extensors with an increase in the vasti muscle forces in early stance and a decrease in hamstring forces at the final swing. The neuropathic patients also redistributed their lower limb muscle forces, increasing the hamstring forces at push off. Finally, although there were no changes in the ankle extensor moment of the DNG patients and there was a decrease in hip flexor moment, contrary to the traditional description of the hip strategy, the analysis of the individual muscle contributions suggested the existence of a hip strategy on a muscular level.

## Additional file

Additional file 1: Table S1. Mean (± 1 standard deviation) of hip, knee and ankle joint maximum flexion, maximum extension and range of motion (ROM) of the control (CG), diabetic (DG) and diabetic neuropathy (DNG) groups during gait cycle. **Figure S1.** Mean (± 1 standard error) of the hip, knee and ankle angular displacement of the control group (CG) in green, diabetic group (DG) in blue and diabetic neuropathy group (DNG) in red throughout the gait cycle. **Table S2.** Mean (standard deviation) of hip, knee and ankle joint moments during the stance phase of gait. **Figure S2.** Mean (± 1 standard error) of the hip, knee and ankle joint moments (normalized by body weight and height) in the control group (CG), diabetic group (DG) and diabetic neuropathy group (DNG) during the gait cycle. **Table S3.** Ankle muscle’s peak force values (mean ± standard deviation) normalized by body weight (BW) of diabetic neuropathy (DNG), diabetic (DG) and control (CG) groups during the gait cycle. **Figure S3.** Mean (± 1 standard error) of the force time series for the knee flexors muscles (bicep femoris long head and semitendinosus), knee extensor muscles (vastus intermedius and rectus femoris), ankle flexor and extensor muscles (extensor hallucis longus and flexor hallucis longus) and ankle evertor muscles (peroneus longus and peroneus brevis) in the control group (CG - green), diabetic group (DG – blue), and diabetic neuropathy group (DNG – red), during the gait cycle. (DOCX 970 kb)

## Abbreviations

CG: Control Group
DG: Diabetic Group
DNG: Diabetic Neuropathy Group
ROM: Range of Motion
