# Supplementary material for: Muscle force distribution of the lower limbs during walking in diabetic individuals with and without polyneuropathy
Source: J Neuroeng Rehabil. 2017 Nov 9;14:111. doi: 10.1186/s12984-017-0327-x (PMC5679149; doi:10.1186/s12984-017-0327-x)
Supplement: Additional file 1: Table S1. — Mean (± 1 standard deviation) of hip, knee and ankle joint maximum flexion, maximum extension and range of motion (ROM) of the control (CG), diabetic (DG) and diabetic neuropathy (DNG) groups during gait cycle. Figure S1. Mean (± 1 standard error) of the hip, knee and ankle angular displacement of the control group (CG) in green, diabetic group (DG) in blue and diabetic neuropathy group (DNG) in red throughout the gait cycle. Table S2. Mean (standard deviation) of hip, knee and ankle joint moments during the stance phase of gait. Figure S2. Mean (± 1 standard error) of the hip, knee and ankle joint moments (normalized by body weight and height) in the control group (CG), diabetic group (DG) and diabetic neuropathy group (DNG) during the gait cycle. Table S3. Ankle muscle’s peak force values (mean ± standard deviation) normalized by body weight (BW) of diabetic neuropathy (DNG), diabetic (DG) and control (CG) groups during the gait cycle. Figure S3. Mean (± 1 standard error) of the force time series for the knee flexors muscles (bicep femoris long head and semitendinosus), knee extensor muscles (vastus intermedius and rectus femoris), ankle flexor and extensor muscles (extensor hallucis longus and flexor hallucis longus) and ankle evertor muscles (peroneus longus and peroneus brevis) in the control group (CG - green), diabetic group (DG – blue), and diabetic neuropathy group (DNG – red), during the gait cycle. (DOCX 970 kb) [file 12984_2017_327_MOESM1_ESM.docx]

**Supplementary Material**

1. **Lower limbs kinematics**

GN individuals had lower knee flexion in the swing phase, particularly during the toe clearance (Table S1 and Figure S1).

**Table S1** - Mean (± 1 standard deviation) of hip, knee and ankle joints maximum flexion, maximum extension and range of motion (ROM) of the control (CG), diabetic (DG) and diabetic neuropathy (DNG) groups during gait cycle.

|  | **Variables** | **CG (n=10)** | **DG (n=10)** | **DNG (n=12)** | **P** |
| --- | --- | --- | --- | --- | --- |
| **Hip** | Maximum flexion stance (°) | 25.2 ±3.6 | 23.6 ±4.5 | 25.3 ±3.0 | 0.314**^1^** |
|  | Maximum extension stance (°) | -14.6 ±5.2 | -17.3 ±5.6 | -13.8 ±4.3 | 0.212^†^ |
|  | Maximum flexion swing (°) | 26.7 ±5.0 | 24.2 ±4.5 | 26.5 ±3.6 | 0.145^†^ |
|  | ROM (°) | 41.3 ±2.8 | 41.4 ±6.0 | 40.4 ±3.6 | 0.605^†^ |
| **Knee** | Initial Flexion (°) | 10.8 ±3.5 | 14.7 ±5.7 | 12.0 ±5.8 | 0.228^†^ |
|  | Maximum flexion swing (°) | 62.5 ±4.1 ^a^ | 60.3 ±5.4 | 58.3 ±5.5 ^a^ | **0.012**^†^ |
|  | ROM (°) | 61.8 ±4.3 | 60.1 ±6.3 | 58.2 ±6.7 | 0.073^†^ |
| **Ankle** | Initial Flexion stance | -4.3 ±4.1 | -2.1 ±2.1 | -2.2 ±2.0 | 0.884^†^ |
|  | Maximum flexion stance (°) | 13.2 ±6.6 | 13.2 ±3.1 | 13.1 ±3.8 | 0.996^†^ |
|  | Maximum extension stance (°) | -7.5 ±6.6 | -4.8 ±6.3 | -5.0 ±5.9 | 0.372^†^ |
|  | Maximum flexion swing (°) | 12.8 ±5.4 | 12.7 ±6.1 | 10.5 ±4.4 | 0.392^†^ |
|  | ROM (°) | 20.7 ±4.9 | 18.0 ±6.2 | 18.2 ±4.3 | 0.112^†^ |

^†^ Univariate ANOVA. ^a,b^ Post-hoc Newman-Keuls ^( a, b^ showed the significantly different pair of values between groups, p<0.05 adopted). Bold p<0.05


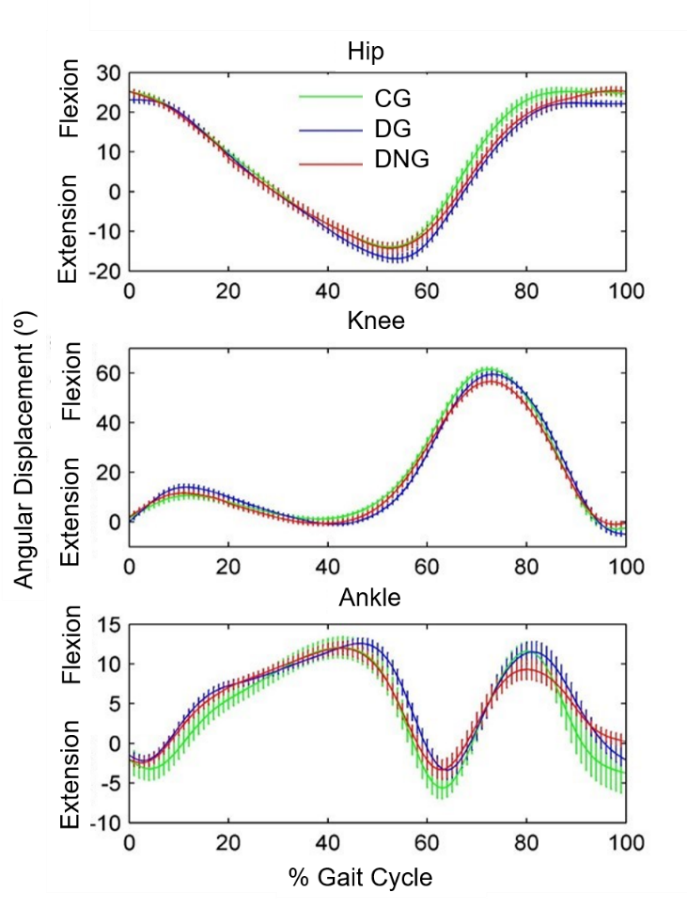


**Figure S1** – Mean ((± 1 standard error) of the hip, knee and ankle angular displacement of the control group (CG) in green, diabetic group (DG) in blue and diabetic neuropathy group (DNG) in red throughout the gait cycle.

Extensão

Extensão

1. **Joint Moments**

The hip flexor moment peak was lower in the DNG compared to CG and DG. DG showed a lower ankle extensor moment compared to CG and DNG (Table 1 and Figure 1).

**Table S2** - Mean (standard deviation) of hip, knee and ankle joint moments during the stance phase of gait.

|  | **Variables** | **CG (n=10)** | **DG (n=10)** | **DNG (n=12)** | **p** |
| --- | --- | --- | --- | --- | --- |
| **Hip** | Peak extensor moment (%BW*height) | - 3.0 (1.5) | - 2.5 (0.8) | - 2.9 (1.0) | 0.520^†^ |
|  | Peak flexor moment (%BW*height) | 4.8 (1.4) ^a^ | 4.8 (1.6) ^b^ | 3.9 (0.9) ^a,b^ | **0.022**^†^ |
| **Knee** | Peak extensor moment (%BW*height) | - 1.8 (1.3) | - 2.0 (0.8) | - 1.7 (0.9) | 0.092^†^ |
|  | Peak flexor moment (%BW*height) | 2.9 (0.9) | 3.4 (0.8) | 3.3 (0.8) | 0.183^†^ |
| **Ankle** | Peak flexor moment (%BW*height) | 0.8 (0.3) | 0.7 (0.3) | 0.7 (0.4) | 0.447^†^ |
|  | Peak extensor moment (%BW*height) | - 8.2 (0.7) ^a^ | - 7.8 (0.7) ^a,b^ | - 8.1 (0.4) ^b^ | **0.042**^†^ |

^†^ Univariate ANOVA. ^a,b^ Post-hoc Newman-Keuls ^( a, b^ showed the significantly different pair of values between groups, p<0.05 adopted). Bold p<0.05


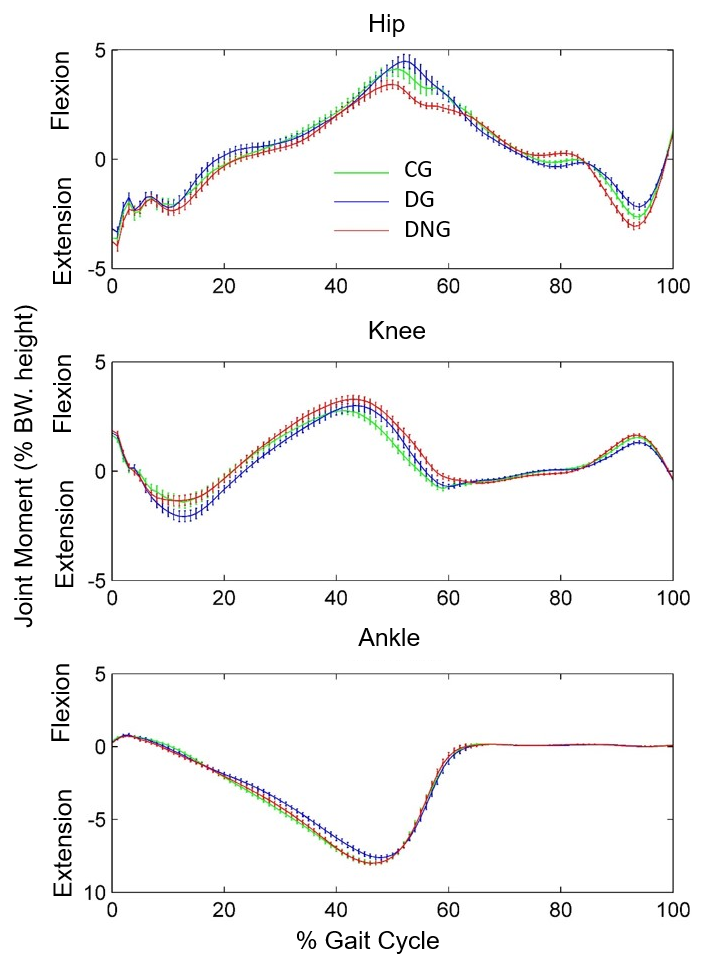


**Figure S2** – Mean (± 1 standard error) of the hip, knee and ankle joint moments (normalized by body weight and height) in the control group (CG), diabetic group (DG) and diabetic neuropathic group (DNG) during the gait cycle.

1. **Muscle Force**

**Table S3** – Ankle muscle’s peak force values (mean ± standard deviation) normalized by body weight (BW) of diabetic neuropathy (DNG), diabetic (DG) and control (CG) groups during the gait cycle.

| **Muscles (% Gait Cycle)** | **Force Peak (% BW)** | | |  |  |
| --- | --- | --- | --- | --- | --- |
|  | **CG^1^ (n=10)** | **DG^2^ (n=10)** | **DNG^3^ (n=12)** | **DCohen** | **p**^†^ |
| **Extensor Hallucis Longus**  **(40 – 80%)** | 0.4±0.1 ^a^ | 0.5±0.3 ^b^ | 0.7±0.4 ^a,b^ | (1x2) 0.477  (1x3) 1.029  (2x3) 0.566 | **0.002**^†^ |
| **Extensor Hallucis Longus**  **(0 – 30%)** | 0.7± 0.2 | 0.6± 0.3 | 1.7± 0.5 | (1x2) -0.392  (1x3) 2.626  (2x3) 2.668 | 0.065 |
| **Flexor Hallucis Longus**  **(0 – 35%)** | 1.3±0.8 | 1.1±0.5 | 1.3±0.5 | (1x2) -0.300  (1x3) 0  (2x3) 0.400 | 0.506^†^ |
| **Flexor Hallucis Longus**  **(35 – 60%)** | 1.8± 0.9 | 0.4± 0.2 | 0.9± 0.7 | (1x2) -2.148  (1x3) -1.116  (2x3) 0.971 | 0.141 |

^†^ ANOVA**,** ^a,b^ Post-hoc Newman-Keuls ^( a, b^ showed the significantly different pair of values between groups, p<0.05 adopted), 1 represent CG, 2 represent DG and 3 represent DNG. Bold (p<0.05).


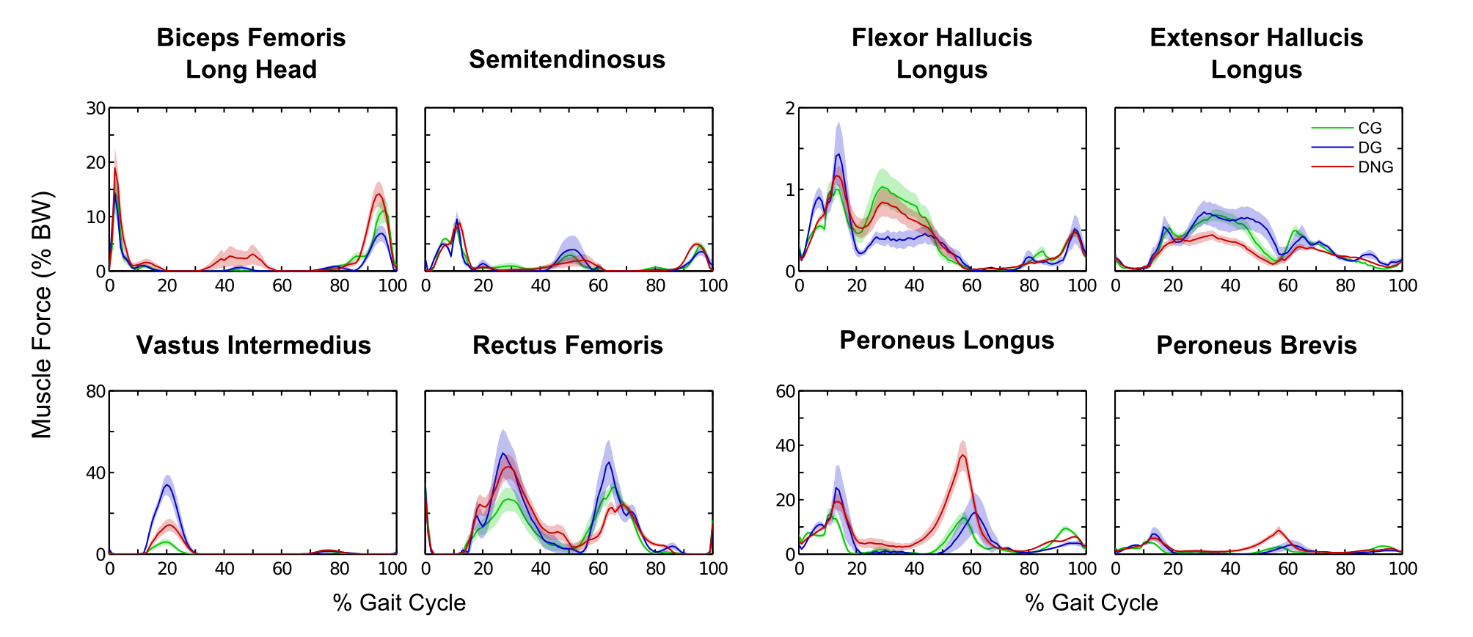


**Figure S3** - Mean (± 1 standard error) of the force time series for the knee flexors muscles (bicep femoris long head and semitendinosus), knee extensor muscles (vastus intermedius and rectus femoris), ankle flexor and extensor muscles (extensor hallucis longus and flexor hallucis longus) and ankle evertor muscles (peroneus longus and peroneus brevis) in the control group (CG - green), diabetic group (DG – blue), and diabetic neuropathic group (DNG – red), during the gait cycle.
